# Personalized Predictions of Therapeutic Hypothermia Outcomes in Cardiac Arrest Patients with Shockable Rhythms Using Explainable Machine Learning

**DOI:** 10.3390/diagnostics15030267

**Published:** 2025-01-23

**Authors:** Chien-Tai Hong, Oluwaseun Adebayo Bamodu, Hung-Wen Chiu, Wei-Ting Chiu, Lung Chan, Chen-Chih Chung

**Affiliations:** 1 Department of Neurology, Taipei Medical University, Shuang Ho Hospital, New Taipei City 235, Taiwan; ct.hong@tmu.edu.tw (C.-T.H.); 11440@s.tmu.edu.tw (W.-T.C.); cjustinmd@gmail.com (L.C.); 2 Department of Neurology, School of Medicine, College of Medicine, Taipei Medical University, Taipei City 110, Taiwan; 3 Taipei Neuroscience Institute, Taipei Medical University, Shuang Ho Hospital, New Taipei City 235, Taiwan; 4 Department of Prevention and Community Health, Milken Institute School of Public Health, The George Washington University, Washington, DC 20052, USA; dr_bamodu@yahoo.com; 5 Directorate of Postgraduate Studies, School of Clinical Medicine, Muhimbili University of Health and Allied Sciences, Ilala District, Dar es Salaam P.O. Box 65001, Tanzania; 6 Ocean Road Cancer Institute, Ilala District, Dar es Salaam P.O. Box 3592, Tanzania; 7 Graduate Institute of Biomedical Informatics, College of Medical Science and Technology, Taipei Medical University, Taipei City 110, Taiwan; hwchiu@tmu.edu.tw; 8 Clinical Big Data Research Center, Taipei Medical University Hospital, Taipei City 110, Taiwan

**Keywords:** artificial neural network, cardiac arrest, therapeutic hypothermia, shockable rhythms, machine learning, Shapley Additive exPlanations, clinical outcome

## Abstract

**Background:** Therapeutic hypothermia (TH) represents a critical therapeutic intervention for patients with cardiac arrest, although treatment efficacy and prognostic factors may vary between individuals. Precise, personalized outcome predictions can empower better clinical decisions. **Methods:** In this multi-center retrospective cohort study involving nine medical centers in Taiwan, we developed machine learning algorithms to predict neurological outcomes in patients who experienced cardiac arrest with shockable rhythms and underwent TH. The study cohort comprised 209 patients treated between January 2014 and September 2019. The models were trained on patients’ pre-treatment characteristics collected during this study period. The optimal artificial neural network (ANN) model was interpretable using the SHapley Additive exPlanations (SHAP) method. **Results:** Among the 209 enrolled patients, 79 (37.80%) demonstrated favorable neurological outcomes at discharge. The ANN model achieved an area under the curve value of 0.9089 (accuracy = 0.8330, precision = 0.7984, recall = 0.7492, specificity = 0.8846) for outcome prediction. SHAP analysis identified vital predictive features, including the dose of epinephrine during resuscitation, diabetes status, body temperature at return of spontaneous circulation (ROSC), whether the cardiac arrest was witnessed, and diastolic blood pressure at ROSC. Using real-life case examples, we demonstrated how the ANN model provides personalized prognostic predictions tailored to individuals’ distinct profiles. **Conclusion:** Our machine learning approach delivers personalized forecasts of TH outcomes in cardiac arrest patients with shockable rhythms. By accounting for each patient’s unique health history and cardiac arrest event details, the ANN model empowers more precise risk stratification, tailoring clinical decision-making regarding TH prognostication and optimizing personalized treatment planning.

## 1. Introduction

Therapeutic hypothermia (TH), also known as targeted temperature management (TTM), is a promising intervention for ameliorating neurologic damage and enhancing prognoses after cardiac arrest [1,2]. However, research on the treatment’s outcomes presents conflicting evidence [3,4,5]. The discrepancies in research findings may result from variations in patient subgroups, study designs, methods used for implementing TH protocols, and assessment time points [6,7,8,9]. Recent meta-analyses indicate TH improves survival and neurological prognosis, specifically in the cardiac arrest subpopulation presenting with initial shockable rhythms [7,8,9]. However, knowledge gaps persist regarding influential factors and tailored predictive models for this group despite the prognostic significance of the rhythms [10,11]. Although studies have explored prognostic factors and developed prediction models for populations undergoing TH [12,13,14,15,16,17,18,19], these tools are not explicitly customized for patients with shockable rhythms. Improving risk stratification in this subgroup requires predictive modeling tailored to their treatment responses and prognostic indicators [7,8,9]. As treatment options expand and evidence highlights the potential benefits of TH for shockable rhythms, accurate prognostic prediction has become crucial for optimizing care and enhancing the efficacy and efficiency of TH utilization. Reliable prognostic predictions enable clinicians to judiciously direct treatments or allocate potentially scarce resources based on personalized risk assessments [13,14,15]. Such predictions also facilitate the prompt deployment of proactive measures when the chances of recovery or survival warrant continued aggressive care [19].

The complexity of care for cardiac arrest patients renders manual elucidation of predictive patterns arduous. Heterogeneous and multidimensional patient data often obscure subtle outcome correlations undetectable by conventional statistics [18,19]. With inherent pattern recognition capabilities, machine learning methods can assimilate intricate combinations of clinical and treatment factors to uncover personalized relationships within high-dimensional data unseen via traditional analysis [11,18,20]. Thus, the ongoing significance of optimizing and refining predictive models, especially for specific patient subgroups, such as those with shockable rhythms, is underscored.

The current study thoroughly examines relevant clinical prognostic factors for patients with cardiac arrest presenting with shockable rhythms and uses an interpretable machine learning strategy that has specifically been designed to predict the neurological outcomes of TH in this patient population. We used pre-TH patient data to establish predictive models for neurological outcomes. Interpretable machine learning techniques were applied to assess the effectiveness, reliability, and clinical significance of the predictive factors. Our modeling aimed to strike a balance between predictive prowess and transparency, elucidating personalized prognostic patterns hidden within heterogeneous treatment data.

## 2. Materials and Methods

### 2.1. Ethics Approval and Consent to Participate

This study was approved by the Joint Institutional Review Board of Taipei Medical University (TMU-JIRB Approval No. N201711046; 19 January 2018). TMU-JIRB granted a waiver for informed consent as this study involved secondary analysis of de-identified electronic medical record data. All procedures were conducted in accordance with the approved study protocol, adhering to standard regulations and the principles outlined in the Declaration of Helsinki.

### 2.2. Participants

This retrospective cohort study analyzed clinical data from the Taiwan Cardiac Arrest Targeted Temperature Management Network (TIMECARD) registry, a nationwide multi-center registry, covering medical records of patients treated at nine medical centers across Taiwan from January 2014 to September 2019. All records were de-identified to ensure confidentiality [21].

For the current study, the inclusion criteria were as follows: (i) age ≥ 18 years, (ii) occurrence of a cardiac event within or outside the hospital, (iii) initial presentation with a shockable cardiac rhythm, (iv) administration of cardiopulmonary resuscitation (CPR) resulting in the return of spontaneous circulation (ROSC), (v) Glasgow Coma Scale (GCS) score of less than 8 or the inability to follow commands after ROSC, and (vi) initiation of TH within 12 h of ROSC.

The exclusion criteria were as follows: (i) uncontrollable bleeding; (ii) impaired consciousness preceding cardiac arrest, indicated by a cerebral performance category (CPC) score of ≥3, irrespective of etiology; (iii) presence of fatal ventricular arrhythmia (tachycardia or fibrillation); (iv) evidence of intracranial hemorrhage; or (v) life expectancy of less than six months. Cases with incomplete data were excluded to maintain data integrity and avoid biases from imputation, ensuring robust model development and validation.

A shockable rhythm refers to pulseless ventricular tachycardia (VT) or fibrillation (VF) observed during resuscitation. Witnessed collapse denotes that an individual observed the onset of a cardiac arrest event. The terms epinephrine dose and amiodarone dose indicate the dosage of these medications administered during the resuscitation.

All eligible patients received therapeutic management according to the Taiwan Society of Emergency and Critical Care Medicine’s scientific statement consensus on TTM [21]. In the present study, TH was implemented through various cooling modalities, including cold saline infusion, surface cooling blankets, intravascular cooling catheters, extracorporeal membrane oxygenation (ECMO), or a combination thereof, to maintain the patient’s body temperature (BT) within a strictly controlled target range. The protocol mandated maintaining core BT at 32–34 °C for a minimum duration of 24 h, followed by controlled rewarming at a rate of 0.25–0.5 °C per hour. Post-rewarming normothermia (36–37 °C) was strictly maintained for 72 h to prevent pyrexia [21]. No patients receiving TTM at normothermia (36 °C) were included in this cohort.

### 2.3. Outcomes

Patients were divided into two groups on the basis of neurological outcomes at the time of discharge. Those with a CPC score of 1 to 2 points, indicating satisfactory cerebral function or moderate disability, were considered to have a favorable neurological prognosis. By contrast, those with a CPC score of 3 to 5 points, indicating severe disability, coma, vegetative state, or brain death, were considered to have an unfavorable prognosis [21,22].

### 2.4. Statistical Analyses

Descriptive statistics were used to summarize the data. Continuous variables are presented as means ± standard deviations, and categorical variables are expressed as numbers and percentages. Pearson’s chi-square test was employed to determine nonrandom associations between pairs of categorical variables across two groups. Between-group comparisons of continuous variables were performed using Student’s *t*-tests. Analysis of variance was used to determine significant differences in comparisons of continuous variables among ≥3 groups. All hypothesis tests were two-sided, and *p* < 0.05 was considered statistically significant. Demographic and clinical characteristics underwent statistical analyses using STATISTICA (version 14.0; TIBCO Software Inc., Tulsa, OK, USA).

### 2.5. Development of Prediction Models

In developing the predictive models, we employed five representative machine learning algorithms—logistic regression (LR), random forest (RF), support vector machine (SVM), eXtreme gradient boosting (XGBoost), and artificial neural network (ANN).

LR, a classical linear model, was adopted for its high interpretability in elucidating individual variable contributions to prognostic outcomes [23,24]. RF was incorporated for its robust handling of multidimensional data and intrinsic resistance to overfitting [25,26]. SVM was chosen for its unique modeling approach, demonstrating notable efficacy with high-dimensional data and limited sample sizes, enhancing methodological diversity, and reducing algorithmic homogeneity [27]. XGBoost, an optimized variant of gradient boosting machines (GBMs), was utilized for its exceptional computational efficiency and built-in regularization mechanisms, effectively addressing overfitting concerns and the demand for more advanced modeling techniques [20,28]. ANNs were leveraged for their excellent capacity to map non-linear relationships and capture complex feature interactions, particularly pertinent to the multi-factorial nature of cardiac arrest prognostication [18,19,29].

### 2.6. Input Features of the Models

All models utilized the 28 variables in Table 1 as input features representing patient characteristics before initiating TH. Categorical variables underwent frequency encoding. Input variables were standardized to ensure similar scales during the model training process.

### 2.7. Algorithm Configuration and Hyperparameter Optimization

We applied a grid search with five-fold cross-validation for each machine learning model to optimize model performance. This approach allowed us to comprehensively explore the hyperparameter space and identify configurations that maximized predictive accuracy [30]. Hyperparameter tuning was performed using five-fold cross-validation, ensuring that the evaluation of each hyperparameter set was based on multiple validation sets, thereby mitigating overfitting and enhancing generalizability. The hyperparameter ranges and selected configurations are detailed in Supplementary Table S1.

### 2.8. Model Evaluation

The generalizability and robustness of the model were assessed through five-fold cross-validation. The dataset was randomly partitioned into five equal subsets. In each iteration, four subsets (80% of the data) were used for model training, while the remaining subset (20%) served as the validation set to evaluate model performance. This process was repeated five times, with each subset serving once as the validation set and four times as part of the training [31]. During each fold, hyperparameters were optimized using grid search applied to the training data, ensuring that the validation set remained independent and unbiased during the tuning process. The mean area under the receiver operating characteristic curve (AUC), alongside accuracy, precision, recall, and specificity, was computed for each iteration. The model achieving the highest mean AUC across all five folds was selected as the final predictive model.

### 2.9. SHapley Additive exPlanations (SHAP)

We used SHAP to provide interpretations of the final model’s features on the global and local scales [32,33,34]. Global interpretation identifies and quantifies each feature’s contribution to the overall predictions, aiding in understanding how features collectively influence the predictions. Local interpretation utilizes SHAP and feature values to visualize the model’s dependency on individual predictions [34,35]. Using real-world examples, we also employ local interpretation by leveraging SHAP to demonstrate how the predictive model’s outcome for a single sample relies on the respective feature values.

We developed all machine learning models and calculated SHAP values using Python version 3.10.9 (Python Software Foundation, Wilmington, DE, USA).

## 3. Results

### 3.1. Cohort Characteristics

**Figure 1:**
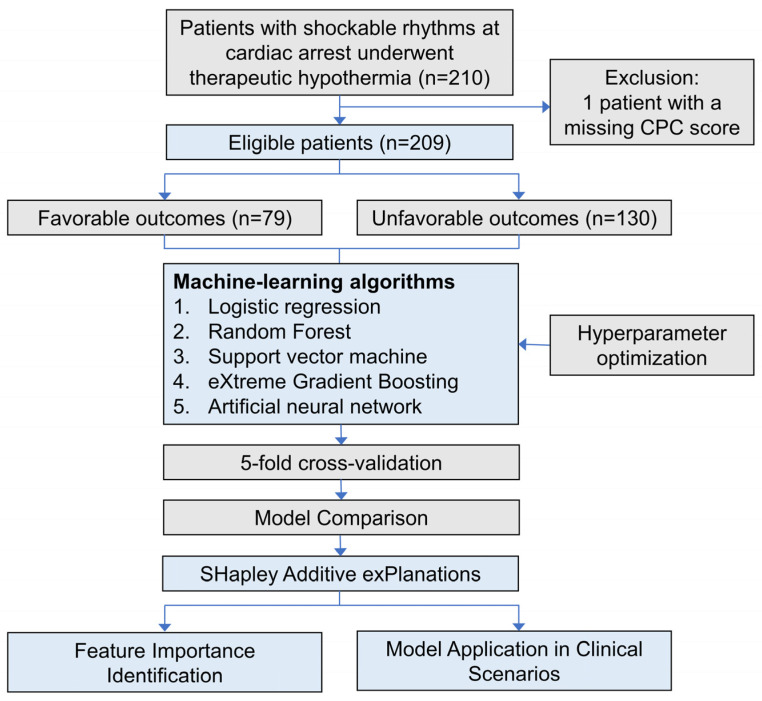
The analytical flowchart employed in the current study. Favorable neurological outcomes were indicated by a CPC score of 1 to 2 points, while unfavorable outcomes were defined as a CPC score of 3 to 5 points. CPC, cerebral performance category.

During the study period, 580 patients were enrolled in the TIMECARD database. Among them, the initial cohort comprised 210 patients with a shockable rhythm and subsequently underwent TH. After excluding one patient without a documented CPC score at discharge to ensure data completeness, 209 patients were included in the assessment (Figure 1). The mean age of these patients was 59.77 ± 14.68 years, and 50 (23.92%) were women. Most of the patients had an out-of-hospital cardiac arrest (OHCA) (86.6%; Table 1); 63 achieved ROSC before arrival at the hospital, and 118 achieved ROSC after admission to the hospital. Upon hospital discharge, 79 patients (37.80%) had favorable neurological outcomes, whereas 130 (62.20%) had unfavorable outcomes. The overall survival rate was 55.50%, with 116 patients surviving. The patients with favorable outcomes tended to be younger and had fewer preexisting illnesses than those with unfavorable outcomes (Table 1).

As illustrated in Table 1, compared with their peers, patients with favorable neurological outcomes were mainly those with witnessed collapse during cardiac arrest, received lower doses of epinephrine and amiodarone during resuscitation, and had more instances of pre-hospital ROSC. Moreover, favorable neurological outcomes correlated with higher BT, systolic blood pressure (SBP), diastolic blood pressure (DBP), and GCS scores at the time of ROSC.

In terms of comorbidities, a history of diabetes mellitus (DM), chronic obstructive pulmonary disease or asthma, coronary artery disease, and malignancy were identified to be associated with unfavorable neurological outcomes (Table 1).

### 3.2. Hypothermia Treatment and Complications

Treatment protocols, including induction, maintenance, and warming duration, were consistent across centers and did not vary between favorable and unfavorable neurological outcomes groups (Table 2). Patients with unfavorable outcomes exhibited higher likelihoods of bleeding, arrhythmias, and seizures compared to those with favorable outcomes.

### 3.3. Predictive Model Development and Performance

**Figure 2:**
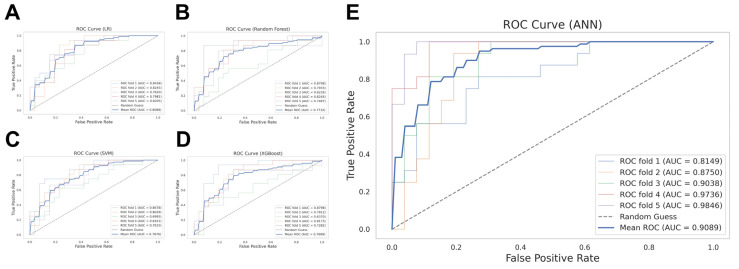
Predictive performance of the machine learning models. Receiver operating characteristic (ROC) curves and area under the curve (AUC) constructed through 5-fold cross-validation for the (**A**) LR, (**B**) random forest, (**C**) SVM, (**D**) XGBoost, and (**E**) ANN. Each point on the mean ROC curve is plotted based on the average true positive rate and false positive rate calculated from each of the five validation folds. Abbreviations: ANN, artificial neural network; AUC, area under the curve; LR, logistic regression; ROC, receiver operating characteristic; SVM, support vector machine; XGBoost, eXtreme gradient boosting.

We constructed machine learning models (LR, RF, SVM, XGBoost, ANN) to predict neurological outcomes using 28 baseline variables (Figure 2). Among these models, the ANN demonstrated the most robust predictive performance, with an AUC of 0.9089, accuracy of 0.8330, precision of 0.7984, recall of 0.7492, and specificity of 0.8846. Table 3 comprehensively compares the predictive performance across the five machine learning algorithms implemented in this study.

**Figure 3:**
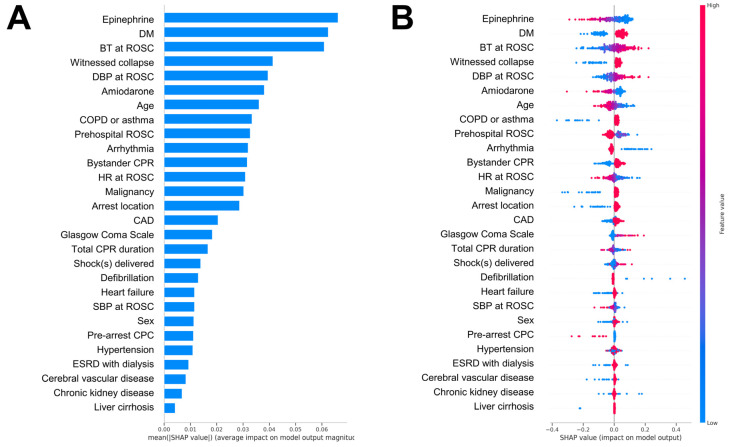
Bar plot and the beeswarm summary plot of the SHAP values of the ANN model. (**A**) The importance of each feature is ranked by the mean absolute SHAP value. (**B**) Effect of each variable on the predictive performance of the model. Different colors indicate the magnitude of variable values, with red indicating higher values and blue indicating lower values. The X-axis presents the effect of different variables on the SHAP values. Abbreviations: ANN, artificial neural network; BT, body temperature; CAD, coronary artery disease; COPD: chronic obstructive pulmonary disease; CPC, cerebral performance category; CPR: cardiopulmonary resuscitation; DBP: diastolic blood pressure; DM: diabetes mellitus; ESRD, end-stage renal disease; HR, heart rate; ROSC, return of spontaneous circulation; SBP: systolic blood pressure; SHAP, SHapley Additive exPlanations.

SHAP analysis for feature contribution assessment identified epinephrine dosage, DM, BT at ROSC, witnessed arrest, and DBP at ROSC as the five most significant predictive parameters (Figure 3A,B). Amiodarone administration and patient age followed, ranking sixth and seventh in importance, respectively.

**Figure 4:**
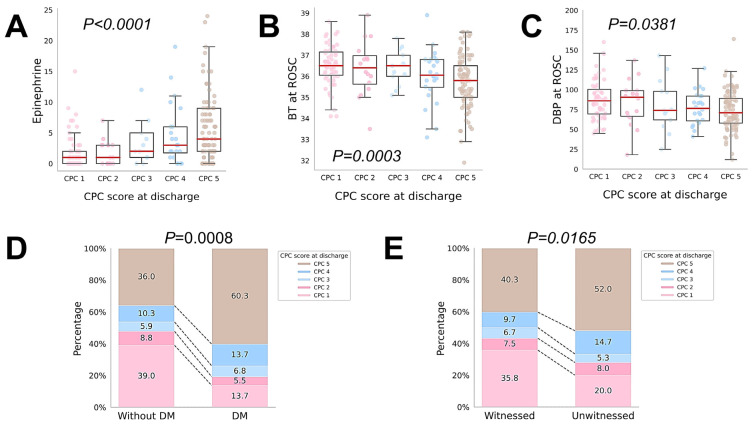
Associations between the top five crucial features and discharge CPC scores at discharge. Continuous variables include the (**A**) dosage of epinephrine, (**B**) BT, and (**C**) DBP at ROSC. Categorical variables include (**D**) diabetes mellitus and (**E**) witnessed collapse. BT, body temperature; CPC, cerebral performance category; DBP, diastolic blood pressure; DM, diabetes mellitus; ROSC, return of spontaneous circulation.

Further validation between the top-five significant features and CPC scores revealed that higher CPC scores at discharge were associated with higher epinephrine doses, lower BT, and lower DBP at ROSC. In addition, patients with diabetes and unwitnessed arrest were associated with higher CPC scores at discharge (Figure 4A–E).

### 3.4. Real-World Model Application

**Figure 5:**
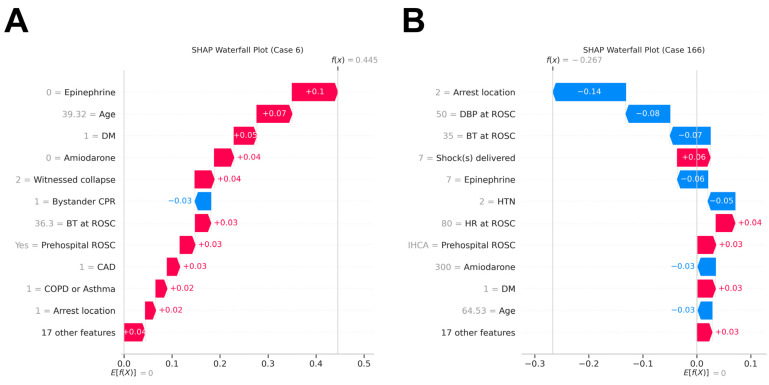
Exploring patient cases: real-life application of explainable machine learning model. We retrospectively employed the SHAP waterfall plot to illustrate the application of the machine learning model in clinical settings for two patients who underwent therapeutic hypothermia. (**A**) A patient with OHCA who had a favorable outcome; (**B**) A patient with IHCA who had a fatal outcome after therapeutic hypothermia. BT, body temperature; CAD, coronary artery disease; COPD: chronic obstructive pulmonary disease; CPR: cardiopulmonary resuscitation; DBP, diastolic blood pressure; DM, diabetes mellitus; HR, heart rate; HTN, hypertension; IHCA, in-hospital cardiac arrest; OHCA, out-of-hospital cardiac arrest; ROSC, return of spontaneous circulation; SHAP, Shapley Additive exPlanations.

We evaluated our model through simulated clinical deployment using two actual cardiac arrest cases from our cohort. Using SHAP visualizations, we retrospectively applied the trained ANN algorithm based on the documented characteristics of a survivor (Figure 5A) and a non-survivor (Figure 5B) after TH.

In the simulation for the survivor, Patient 1 (Figure 5A), a 39-year-old man with no preexisting systemic illnesses, experienced a witnessed OHCA in September 2014. He received 24 min of CPR, including three defibrillation attempts without the use of epinephrine or amiodarone. Upon achieving pre-hospital ROSC, his vitals were as follows: blood pressure, 134/89 mmHg; heart rate, 110 beats per minute; and BT, 36.3 °C. The patient underwent TH using an icy catheter with a 2 h induction, followed by 24 h maintenance and a 13.7 h rewarming. Despite complications of hypokalemia and hypoglycemia, he was discharged after 24 days with a favorable CPC score of 1. The simulation indicated a shift from an initially neutral prognostic baseline to a favorable outcome, attributed to patient-specific factors such as his young age, absence of medication during CPR, and higher BT upon ROSC, and the prediction aligned with the patient’s actual positive outcomes.

In the simulation for the non-survivor, Patient 2 (Figure 5B), a 64-year-old man with a history of hypertension, experienced witnessed IHCA in May 2017. The arrest began with a shockable rhythm, prompt bystander CPR, including seven defibrillator shocks, and administration of 7 mg of epinephrine and 300 mg of amiodarone. Upon ROSC, his vital parameters were as follows: blood pressure, 70/50 mmHg; heart rate, 80 beats per minute; BT, 35 °C; and GCS, 3. For the patient, the model’s prediction trajectory shifted from neutral toward a poor prognosis driven by recognized detriments. This prediction was consistent with the patient’s actual outcome. On the same day of arrest, the patient underwent TH with ECMO; the patient experienced post-TH complications of arrhythmia and hypokalemia, and passed away two days later.

## 4. Discussion

This study conducted an in-depth analysis of patients who experienced cardiac arrest with shockable rhythm and subsequently underwent TH. By scrutinizing demographic data, characteristics of cardiac arrest events, interventions during treatment, and post-treatment outcomes, we offer a comprehensive understanding of this specific patient cohort. Our ANN model accounted for patients’ unique health histories, characteristics of cardiac arrest events, and resuscitation details, delivering precise individualized risk stratification. The post-cross-validation AUC value of 0.9089 demonstrated its excellent predictive performance.

The sophisticated pattern recognition capabilities of machine learning empower comprehensive analysis of intricate combinations of variables, uncovering personalized correlations. In contrast to traditional statistical methods, machine learning algorithms excel in detecting multivariable interactions, providing enhanced predictive accuracy through integrated parameter analysis, which is particularly pertinent in cardiac arrest patients with numerous clinical parameters. By quantifying variable contributions through SHAP values, we strengthen model transparency regarding how unique parameters influence prognostic estimations. These parameters included epinephrine dose during resuscitation, DM, BT at ROSC, witnessed arrest, and DBP at ROSC. Furthermore, amiodarone dosage and age ranked as the sixth and seventh most influential variables, respectively, reflecting the complex multivariable interactions affecting prognostic outcomes.

Notably, diabetes emerged as a more significant prognostic indicator than age in our analysis. While age is established as a critical determinant of neurological recovery following cardiac arrest and TH [36,37], our cohort demonstrated significantly younger age in patients with favorable outcomes versus poor outcomes (54.95 ± 15.30 vs. 62.70 ± 13.55 years, *p* = 0.0003, Table 1). The elevated prognostic significance of DM may be attributed to its deleterious effects on metabolic homeostasis, endothelial function, and microcirculatory integrity [38,39]. Moreover, potential synergistic interactions between diabetes and other clinical parameters, including increased epinephrine requirements and hypotension, may amplify its adverse impact, potentially explaining its superior ranking relative to age. These findings underscore the necessity for individualized risk assessment and treatment strategies, particularly for diabetic or elderly patient populations. As demonstrated in Figure 5, machine learning models excel in evaluating outcomes based on each patient’s unique characteristics. This individualized predictive modeling may facilitate more precise treatment plan formulation.

As an advanced tool, machine learning effectively handles complex datasets and captures non-linear relationships [11,18,19,20,40] Our study employs interpretable algorithmic techniques and has been validated across multiple centers to ensure generalizability in diverse clinical settings. We emphasize the impact of individual patient characteristics on predicted outcomes, demonstrating the practical utility of ANN in forecasting neurological outcomes while providing tailored explanations of variable importance and prediction logic.

TH has emerged as a pivotal therapeutic intervention in our cohort, comprising patients with shockable rhythms, integrated into contemporary post-resuscitative protocols at our study centers. This standardized approach allowed for the mitigation of post-cardiac arrest cellular injury and improved recovery prospects [1,2,21]. Crucially, by elucidating TH’s role within the current cohort, we contextualized the application of predictive analytics in a real-world clinical scenario, advancing the understanding of such models’ utility and generalizability in enhancing patient care. Precise predictions can discriminate responders to treatment from those whose condition may be exacerbated, guiding the early initiation of aggressive interventions to optimize survival and quality of life. It is essential to emphasize that the proposed model serves primarily to identify patients requiring enhanced monitoring and additional supportive interventions during TH rather than to determine eligibility for the therapy itself. This distinction is crucial, as current guidelines recommend considering TH for all patients who meet established criteria. The model aims to enable early recognition of high-risk patients who may benefit from more intensive care measures concurrent with TH, such as advanced hemodynamic monitoring, tailored pharmacologic adjustments, or specialized neurological interventions.

In resource-intensive critical care settings, early risk stratification through predictive analytics can facilitate the proactive implementation of complementary therapies and optimize the level of monitoring while maintaining TH as the standard of care. This strategy aligns with the principle of providing timely and aggressive interventions to improve outcomes while ensuring that all eligible patients receive appropriate temperature management [1,21,41]. In resource-constrained resuscitative care settings, reliable neurological prognostic assessments enable the rational distribution of resources to the most vulnerable patients, addressing challenges posed by medical resource scarcity. By prioritizing high-risk patients for intensive monitoring and advanced interventions, this approach mitigates the risk of irreversible neurological damage and maximizes therapeutic resource utilization [14].

The developed model functions as a clinical decision-support tool to enhance patient care. Through precise risk stratification and tailored supportive measures, it optimizes treatment strategies within the established framework of post-cardiac arrest care, with TH remaining a fundamental component.

The present model incorporates both OHCA and IHCA patients, accounting for the potential influence of different cardiac arrest locations on relevant factors and outcomes. This inclusive design enhances representativeness and generalizability, rendering the model applicable across diverse healthcare settings. A literature review indicates that machine learning applications in predicting TH outcomes remain underexplored [11,14,15,16,17,19,29,42]. Our assessment focuses on patients presenting with shockable rhythms, a methodology substantiated by extensive evidence demonstrating superior neurological outcomes following TH in this subgroup [7,8,9]. This selective inclusion reduces population heterogeneity by excluding non-shockable rhythm patients—who typically have poorer prognoses—thereby enhancing model precision and clinical utility.

Previous investigations employing machine learning for outcome prediction in post-arrest patients undergoing TH have consistently highlighted initial rhythm as a key prognostic factor. Johnsson et al. [42] and Andersson et al. [29] applied ANN and LR to 932 patients, underscoring rhythm as a dominant predictor. Kołtowski et al. [16] corroborated these findings through a multivariable LR-based risk score utilizing Polish Hypothermia Registry data (n = 376). In Asian cohorts, Chung et al. [11] validated rhythm’s prognostic significance in IHCA patients (n = 796, external validation n = 108), while Chou et al. demonstrated concordant results utilizing an ANN-augmented CASPRI score (n = 570). Lin et al. [17] reinforced rhythm’s predictive role by constructing a stepwise multiple LR model for OHCA patients (n = 408).

While acknowledging the established prognostic value of initial cardiac arrest rhythm, excessive emphasis may obscure other critical clinical variables, including diabetes, post-resuscitation hemodynamic parameters, and pharmacological interventions. Incorporating heterogeneous rhythm types within a single model may introduce methodological bias, potentially compromising risk assessment accuracy within specific patient subgroups.

Our analysis, by exclusively focusing on shockable rhythm patients, highlights witnessed arrest, BT at ROSC, DM, and epinephrine dosage as critical predictors of poor neurological outcomes. This refinement distinguishes our study from previous investigations and uncovers prognostic determinants underemphasized in prior models. Through interpretative methods and real-world application, we enhance transparency and demonstrate the clinical relevance of predictive features, bridging gaps left by previous research. From a research perspective, the current model provides a framework for identifying and quantifying the contribution of individual patient characteristics to TH outcomes. This framework can guide future research endeavors in exploring the underlying mechanisms and pathways influencing treatment responses, deepening our understanding of the complex interplay between patient profiles and TH efficacy. From a clinical perspective, this approach aligns with the growing emphasis on precision medicine, wherein treatments are tailored to individual patient characteristics. By elucidating the specific factors driving outcomes, our model can inform the development of more personalized TH protocols and aid in optimizing treatment strategies, resource allocation, and patient counseling.

This study has several limitations. First, despite being a multi-center study, the small number of cases with shockable rhythms considered in the study may have limited the comprehensiveness of our analysis of patients with different characteristics, potentially limiting the generalizability and applicability of our results. Second, our study employed five-fold cross-validation without incorporating an independent test dataset, thereby hindering the definitive assessment of the model’s practical applicability. Forthcoming research should further utilize independent validation datasets to verify the established model’s performance and robustness across diverse clinical environments, strengthening its potential for broader clinical application. Third, our study cohort consisted exclusively of patients who underwent TH without including those managed under normothermia. This selective criterion precludes the model’s ability to differentiate between patients who benefit from TH or non-TH approaches. This limits the model’s applicability in guiding the decision between TH and normothermia. Fourth, our registry records did not provide detailed medical history before the cardiac arrest for patients with IHCA, potentially preventing us from gaining a comprehensive understanding of patient conditions. Another notable limitation pertains to the practical operationalization of our model in acute resuscitation scenarios. The requirement to input 28 patient variables manually poses challenges in time-sensitive situations. Future efforts should streamline data acquisition through electronic health record integration, dedicated software interfaces, and leveraging emerging technologies like wearable devices and sensor networks for automated variable collection.

## 5. Conclusions

This research delves into patients who experienced cardiac arrest with shockable rhythms and undergone TH, focusing on the interconnections between individual risk factors, the cardiac arrest event, resuscitation processes, complications, and neurological outcomes. Our neural-network-based model demonstrated robust predictive performance, while interpretable machine learning techniques enabled the quantification of individual feature contributions, enhancing our understanding of neurological outcome prognostication. Crucially, this predictive model serves as a complementary decision tool for identifying patients who may benefit from intensified monitoring and adjunctive interventions during TH. This aligns with current guidelines recommending TH as a viable post-resuscitative care intervention for eligible patients. The model facilitates early risk stratification, enabling proactive administration of additional supportive measures for high-risk patients while maintaining TH as the cornerstone therapy. Through specific case applications, we demonstrated the model’s practical utility in clinical settings. Our results underscore the significance of individual variability and distinct features in formulating personalized treatment plans, providing substantial insights into machine learning’s immense capabilities in advancing precision medicine when applied judiciously. Simultaneously, it offers tangible references and insights for implementing personalized therapeutic approaches during TH for shockable cardiac arrest patients, fostering improved shared decision-making and seamless integration into clinical workflows by allowing comprehension of the underlying rationale for predictions.

## Figures and Tables

**Table 1:** Baseline demographic characteristics of patients before receiving therapeutic hypothermia stratified by neurologic outcome at discharge.

Variables	Whole Cohort	Neurological Outcomes		*p*-Value
		Favorable	Unfavorable	
Number of patients	209	79	130	
Age (years)	59.77 ± 14.68	54.95 ± 15.30	62.70 ± 13.55	0.0003 ***
Female, n (%)	50 (23.92)	16 (20.25)	34 (26.15)	0.3323
Pre-arrest CPC score	1.07 ± 0.29	1.01 ± 0.11	1.11 ± 0.36	0.0055 **
Arrest location, n (%)				0.1334
OHCA	181 (86.60)	72 (91.14)	109 (83.85)	
IHCA	28 (13.40)	7 (8.86)	21 (16.15)	
Witnessed collapse, n (%)	175 (83.73)	74 (93.67)	101 (77.69)	0.0024 **
Bystander CPR, n (%)	141 (67.46)	59 (74.68)	82 (63.08)	0.0825
Defibrillation, n (%)	203 (97.13)	76 (96.20)	127 (97.69)	0.5317
Shock(s) delivered	2.46 ± 2.30	2.24 ± 1.63	2.59 ± 2.63	0.2340
Epinephrine dose (mg)	3.97 ± 4.70	1.76 ± 2.64	5.32 ± 5.16	<0.0001 ***
Amiodarone dose (mg)	135.42 ± 212.78	89.24 ± 160.26	163.48 ± 235.32	0.0073 **
CPR duration (min)	26.26 ± 19.74	23.25 ± 21.04	28.08 ± 18.75	0.0958
Pre-hospital ROSC, n (%)				0.0052 **
Yes	63 (30.14)	34 (43.04)	29 (22.31)	
No	118 (56.46)	38 (48.10)	80 (61.54)	
IHCA	28 (13.40)	7 (8.86)	21 (16.15)	
SBP at ROSC (mmHg)	128.3 ± 37.1	135.20 ± 35.87	124.16 ± 37.36	0.0351 *
DBP at ROSC (mmHg)	79.38 ± 25.53	86.14 ± 25.94	75.27 ± 24.47	0.0031 **
HR at ROSC (bpm)	101.56 ± 29.39	104.00 ± 31.23	100.08 ± 28.24	0.3629
BT at ROSC (℃)	36.07 ± 1.19	36.49 ± 1.01	35.82 ± 1.23	<0.0001 ***
Glasgow Coma Scale	3.67 ± 1.40	4.05 ± 1.66	3.45 ± 1.18	0.0053 **
Co-morbidities, n (%)				
Diabetes mellitus	73 (34.93)	14 (17.72)	59 (45.38)	<0.0001 ***
Hypertension	114 (54.55)	41 (51.90)	73 (56.15)	0.5491
Coronary artery disease	75 (35.89)	21 (26.58)	54 (41.54)	0.0288 *
Heart failure	37 (17.70)	11 (13.92)	26 (20.00)	0.2645
Arrhythmia	28 (13.40)	12 (15.19)	16 (12.31)	0.5531
COPD or asthma	16 (7.66)	2 (2.53)	14 (10.77)	0.0299 *
Chronic kidney disease	20 (9.57)	5 (6.33)	15 (11.54)	0.2145
ESRD on hemodialysis	19 (9.09)	4 (5.06)	15 (11.54)	0.1144
Liver cirrhosis	2 (0.96)	0 (0)	2 (1.54)	0.2680
Previous cerebral vascular disease	14 (6.70)	3 (3.80)	11 (8.46)	0.1909
Malignancy	21 (10.05)	3 (3.80)	18 (13.85)	0.0191 *

Abbreviations: BT, body temperature; COPD, chronic obstructive pulmonary disease; CPC, cerebral performance category; CPR, cardiopulmonary resuscitation; DBP; diastolic blood pressure; ESRD, end-stage renal disease; HR, heart rate; IHCA, in-hospital cardiac arrest; SBP, systolic arterial pressure; OHCA, out-of-hospital cardiac arrest; ROSC, return of spontaneous circulation. All *p*-values represent comparisons between the favorable and unfavorable neurological outcome groups. * *p* < 0.05; ** *p* < 0.01; *** *p* < 0.001.

**Table 2:** Characteristics of the patients with shockable rhythm during and after therapeutic hypothermia.

Variables	Whole Cohort	Neurological Outcomes		*p*-Value
		Favorable	Unfavorable	
Number of patients	209	79	130	
Maintenance mode, n (%)				0.2990
Arctic Sun cold blanket	94 (44.98)	40 (50.63)	54 (41.54)	
Traditional cold blanket	73 (34.93)	27 (34.18)	46 (35.38)	
ECMO	33 (15.79)	8 (10.13)	25 (19.23)	
Icy catheter	9 (4.31)	4 (5.06)	5 (3.85)	
Induction duration (h)	6.93 ± 6.97	6.81 ± 6.10	7.01 ± 7.48	0.8298
Maintenance duration (h)’	21.88 ± 10.15	22.22 ± 9.93	21.66 ± 10.33	0.7070
Rewarming_duration (hr)	18.55 ± 18.29	16.55 ± 10.90	19.98 ± 22.07	0.1605
Complications, n (%)				
Bleeding	65 (31.10)	17 (21.52)	48 (36.92)	0.0197 *
Arrhythmia	97 (46.41)	29 (36.71)	68 (52.31)	0.0283 *
Serious infection	69 (33.01)	20 (25.32)	49 (37.69)	0.0651
Seizure	52 (24.88)	10 (12.66)	42 (32.31)	0.0014 **
Hypokalemia	144 (68.90)	62 (78.48)	82 (63.08)	0.0197 *
Hypoglycemia	16 (7.66)	6 (7.59)	10 (7.69)	0.9795
Survival duration (days)	23.61 ± 22.39	23.71 ± 21.02	23.54 ± 23.26	0.9576
CPC score at discharge	3.33 ± 1.76	1.20 ± 0.40	4.62 ± 0.66	<0.0001 ***
Survival to discharge, n (%)	116 (55.50)	79 (100)	37 (28.46)	<0.0001 ***

Abbreviations: CPC, cerebral performance category; ECMO, extracorporeal membrane oxygenation. All *p*-values represent comparisons between the favorable and unfavorable neurological outcome groups. * *p* < 0.05; ** *p* < 0.01; *** *p* < 0.001.

**Table 3:** Comparing machine learning models to predict outcomes in cardiac arrest patients with shockable rhythms treated with therapeutic hypothermia.

Model	AUC	Accuracy	Precision	Recall	Specificity
LR	0.8088 (0.77, 0.85)	0.723 (0.68, 0.78)	0.6769 (0.57, 0.78)	0.5833 (0.49, 0.68)	0.8231 (0.74, 0.91)
Random forest	0.7732 (0.65, 0.89)	0.7081 (0.66, 0.75)	0.6654 (0.58, 0.75)	0.4683 (0.34, 0.60)	0.8538 (0.79, 0.92)
SVM	0.7876 (0.71, 0.87)	0.7417 (0.69, 0.79)	0.7301 (0.61, 0.85)	0.5325 (0.37, 0.70)	0.8692 (0.78, 0.96)
XGBoost	0.7689 (0.65, 0.88)	0.7033 (0.67, 0.74)	0.6822 (0.57, 0.79)	0.4175 (0.38, 0.45)	0.8769 (0.81, 0.94)
ANN	**0.9089** (0.82, 1.00)	**0.8330** (0.75, 0.92)	**0.7984** (0.72, 0.88)	**0.7492** (0.55, 0.95)	**0.8846** (0.83, 0.94)

The highest mean value of the predictive metric among the five machine learning models is shown in bold. Numbers in parentheses represent 95% Confidence Intervals (CI). Abbreviations: ANN, artificial neural network; AUC, area under the receiver operating characteristic curve; LR, logistic regression; SVM, support vector machine; XGBoost, eXtreme gradient boosting.

## Data Availability

For ethical and privacy considerations, as mandated by the Joint Institutional Review Board of Taipei Medical University and per relevant regulations, we are unable to publicly share the raw data used in this study. A de-identified and aggregated summary of the data that support the findings presented in this research can be made available upon reasonable request to the corresponding author, and subject to approval by the Research Ethics Review Committee.

## Supplementary Materials

The following supporting information can be downloaded at: https://www.mdpi.com/article/10.3390/diagnostics15030267/s1, Table S1: The hyperparameter tuning setup for machine learning models in the current study.

## Abbreviations

The following abbreviations are used in this manuscript:

ANN	Artificial neural network
AUC	Area under the curve
BT	Body temperature
CPC	Cerebral performance category
CPR	Cardiopulmonary resuscitation
DBP	Diastolic blood pressure
DM	Diabetes mellitus
IHCA	In-hospital cardiac arrest
LR	Logistic regression
OHCA	Out-of-hospital cardiac arrest
RF	Random forest
ROC	Receiver operating characteristic
ROSC	Return of spontaneous circulation
SBP	Systolic blood pressure
SHAP	SHapley Additive exPlanations
SVM	Support vector machine
TH	Therapeutic hypothermia
VF	Ventricular fibrillation
VT	Ventricular tachycardia
XGBoost	eXtreme gradient boosting
