# Supplementary material for: Personalized Predictions of Therapeutic Hypothermia Outcomes in Cardiac Arrest Patients with Shockable Rhythms Using Explainable Machine Learning
Source: Diagnostics (Basel). 2025 Jan 23;15(3):267. doi: 10.3390/diagnostics15030267 (PMC11817524; doi:10.3390/diagnostics15030267)
Supplement: Supplementary file 1 [file diagnostics-15-00267-s001.zip › diagnostics-3411722-supplementary.pdf]

Supplementary Table S1. The hyperparameter tuning setup for machine learning models in the current study

Algorithm	Hyperparameters
Logistic regression	**C**: Regularization parameter, range: 0.001, 0.01, 0.1, 1, 5, 10.<br>**penalty**: L1 regularization and L2 regularization.
Random forest	**n_estimators**: Number of decision trees, range: 100, 200, 300, 400, 500.<br>**max_depth**: Maximum depth of the trees, range: None, 10, 20, 30, 40, 50.<br>**min_samples_split**: Minimum number of samples required to split an internal node, range: 2, 3, 4, 5, 10.<br>**min_samples_leaf**: Minimum number of samples required at a leaf node, range: 1, 2, 3, 4.<br>**max_features:** Maximum number of features to consider for each split, range: 'sqrt', 'log2'.
Support vector machine	**C**: Regularization parameter, range: 0.1, 1, 5, 10.<br>**kernel**: Kernel function type: radial basis function, 'rbf'.<br>**gamma**: Scale parameter for the kernel function, range: 'scale', 'auto', 0.1, 1.
eXtreme Gradient Boosting	**learning_rate**: Learning rate, range: 0.001, 0.01, 0.05, 0.1, 0.2.<br>**max_depth**: Maximum depth of a tree, range: 2, 3, 4, 5, 6.<br>**n_estimators**: Number of boosting rounds, range: 100, 200, 300, 400.<br>**subsample**: Subsample ratio of the training instances, range: 0.7, 0.8, 0.9, 1.0.<br>**colsample_bytree**: Subsample ratio of columns when constructing each tree, range: 0.5, 0.6, 0.7, 0.8.<br>**min_child_weight**: Minimum sum of weights required in a leaf node, range: 1, 2, 3, 4.<br>**gamma**: Minimum loss reduction needed to make a further partition on a leaf node, range: 0, 0.1, 0.2.
Artificial neural network	**Input Layer**: The number of neurons equals the number of features.<br>**Hidden Layer**: The number of neurons, range: 2 to 128, with ReLU activation function,<br>**Output Layer**: 1 neuron with Sigmoid activation function, binary classification.<br>**loss function**: binary cross-entropy<br>**optimizer**: Optimizer for weight optimization, range: 'adam', 'sgd', 'rmsprop' with default learning rates.<br>**batch_size**: Batch size for training, range: [8, 16, 32, 64].<br>**epochs**: Number of training epochs, range: [10, 20, 30, 40, 50].
